# Novel ubiquitination-related biomarkers for Crohn’s disease identified by multi-omics study and experimental validation

**DOI:** 10.3389/fimmu.2025.1687606

**Published:** 2025-12-05

**Authors:** Jia Hu, Kai Nie, Xiaoyan Wang

**Affiliations:** Department of Gastroenterology, The Third Xiangya Hospital, Central South University, Changsha, Hunan, China

**Keywords:** Crohn’s disease, ubiquitination, IFITM3, PSMB9, TAP1

## Abstract

**Background:**

Ubiquitination plays a critical role in the onset and progression of Crohn’s disease (CD). Nevertheless, the role of ubiquitination-related genes (URGs) in CD remains incompletely characterized. This study aimed to identify URGs with diagnostic value in patients with CD and construct a URGs-based diagnostic model for CD.

**Methods:**

Single-cell and bulk RNA sequencing datasets related to CD were retrieved from the Gene Expression Omnibus (GEO) database. Single-cell analysis was conducted to characterize cell subsets associated with ubiquitination processes. CellChat was employed to elucidate potential intercellular communication networks, providing insights into the signaling interactions among different cell subsets. Furthermore, High-dimensional weighted gene co-expression network analysis (hdWGCNA) was performed to identify gene modules significantly correlated with ubiquitination. To screen for ubiquitination-related genes with diagnostic potential, we integrated genes from the identified hdWGCNA modules with differentially expressed genes (DEGs) between CD patients and healthy controls. Subsequently, XGBoost was utilized to further refine and identify core genes. These core genes were then used to construct a gene-based diagnostic model for CD. Finally, the expression levels of the core genes were experimentally validated in both *in vitro* cell models and human tissue biopsy specimens.

**Results:**

We identified IFITM3, PSMB9, and TAP1 as core ubiquitination-related genes in CD patients. The diagnostic model constructed based on these core genes showed remarkable accuracy, with the area under the curve consistently exceeding 0.9. Three core genes correlated significantly with activated immune cells in the inflammatory microenvironment and showed positive correlations with immune checkpoints like CD40, CD80, and CD274. Levels of IFITM3, PSMB9, and TAP1 were significantly elevated in LPS and INF-γ-induced THP-1 cells. Elevated expression of TAP1 and PSMB9 was also confirmed in tissue biopsy specimens, demonstrating the potential of these genes as diagnostic biomarkers.

**Conclusion:**

Ubiquitination-related genes IFITM3, PSMB9, and TAP1 are diagnostic markers for CD. The model based on these three genes offers valuable insights for disease diagnosis and treatment, which facilitates clinical decision-making.

## 1 Introduction

Crohn’s disease (CD) is a subtype of inflammatory bowel disease (IBD), which belonging to the chronic inflammatory intestinal disorder. The pathogenesis of IBD involves the complex interplay between immune, gut microbiota, genetic, and environmental factors (1). The prevalence of IBD exhibits significant geographic variation, with particularly high rates observed in European countries (2). Germany reports a CD prevalence of 322 cases per 100,000 population, while Canada documenting a CD prevalence of 319 per 100,000 (2). Notably, the incidence of IBD has increased markedly in emerging industrialized nations since 1990 (2). Given these epidemiological patterns, the identification of reliable diagnostic biomarkers and elucidation of pathogenic mechanisms in IBD have emerged as current research focus.

Ubiquitination, a critical post-translational modification, governs essential biological processes including proteasomal degradation, signal transduction modulation, gene expression regulation, inflammation and immune process (3). Dysregulation of this process contributes to various pathologies such as cancers, autoimmune disorders, and inflammatory diseases (4). This biological process is mediated through the regulation of the ubiquitin-proteasome system. The ubiquitination cascade comprises three enzymatic stages (5). E1 ubiquitin-activating enzymes initiate the process by transferring activated ubiquitin to E2 conjugating enzymes, which subsequently cooperate with E3 ligases to mediate substrate-specific ubiquitin conjugation. This modification determines the processes such as the proteasomal degradation of substrates, autophagic clearance, or the activation of inflammatory pathways (6). Deubiquitinating enzymes exert an antagonistic effect by removing ubiquitin. Emerging research highlights the pivotal role of ubiquitination and deubiquitination imbalance in IBD pathogenesis. Notably, the E3 ligase ankyrin repeat and SOCS box-containing protein 3 demonstrates marked upregulation in the inflamed intestinal tissues of patients with IBD, driving intestinal dysbiosis and amplifying colonic inflammation through NF-κB pathway activation (7). Ubiquitin-specific proteases are the largest family of deubiquitinating enzymes. USPs regulate IBD through multiple mechanisms, including the NF-κB and TGF-β pathways, and are closely associated with intestinal barrier defects and immune imbalance (8). Moreover, OTUD5 is a deubiquitinating enzyme that is overexpressed in both CD and UC, and it is involved in the amplification of abnormal cytokine responses in IBD (9). Therefore, identifying differentially expressed ubiquitination-related genes (URGs) may lead to new biomarkers for diagnosis and treatment.

In this study, we integrated single-cell sequencing datasets and gene expression datasets to screen ubiquitination-related genes with diagnostic value for CD. Our investigation revealed the roles of IFITM3, PSMB9, and TAP1 in CD diagnosis, and the model constructed based on these genes may represent a novel strategy for identifying patients with CD. We found that the three core genes were significantly correlated with activated immune cells in the inflammatory microenvironment and they exhibited positive correlations with immune checkpoints including CD40, CD80, and CD274 and so on.

## 2 Methods

### 2.1 Patients

The colon biopsy samples used in this study were obtained from patients aged 18 to 60 years old who were newly diagnosed with CD during colonoscopy at the Third Xiangya Hospital of Central South University. Intestinal tissue specimens from the inflamed intestines of CD patients were collected, and intestinal polyp specimens were collected as controls. **Supplementary Material S1** presented the baseline characteristics of involved patients. There were no statistically significant differences among the groups in terms of age, sex. None of them have received treatment. All human studies have been approved by the Ethics Committee of the Third Xiangya Hospital (Approval Number: 21138). All patients included in the study signed a complete informed consent form, and the sample collection process was carried out in accordance with the Declaration of Helsinki.

### 2.2 Datasets and preprocessing

The single-cell sequencing dataset GSE134809, comprising 11 pairs of involved and uninvolved ileal samples from CD patients, was retrieved from the Gene Expression Omnibus (GEO, URL: https://www.ncbi.nlm.nih.gov/geo/). This analysis also incorporated GEO gene expression profile datasets GSE75214, GSE179285, and GSE95095. Dataset GSE75214, containing 59 CD colonic tissues and 22 healthy controls, served as the training set. Dataset GSE179285 (47 CD colonic tissues and 31 healthy controls) and GSE95095 (24 CD colonic tissues and 12 healthy controls) were selected as validation sets. Detailed information was presented in **Supplementary Material S2**.

For the GSE134809 single-cell dataset, quality control was performed to eliminate low-quality data according to the following filtering criteria: (1) Remove genes expressed in fewer than 3 cells. (2) Remove cells expressing fewer than 300 genes. (3) Only include cells where the expression proportion of mitochondrial genes is below 15%, that of ribosomal genes is above 3%, and that of hemoglobin genes is below 0.1%. (4) Filter out the housekeeping gene MALAT and mitochondrial genes. The scRNA-seq data were normalized using the “LogNormalize” method. The top 1500 highly variable genes were screened using the “FindVariableFeatures” function. We reduced the dimensionality of the scRNA-seq data based on the “RunPCA” function and selected the first 50 principal components for further analysis. All cells were clustered using the “FindNeighbors” function of the “Seurat” R package. The “RunUMAP” function was used to visualize the clustering results. Cells were manually annotated based on the reported literature (10).

### 2.3 Analysis of intercellular communication

The CellChat software package (version 1.6.1) was utilized to explore the basic processes of intercellular communication and the ligand-receptor pairs between cell clusters (11). The netVisual_circle function was employed to intuitively display the quantity and intensity of communication between target cell clusters and other cell clusters, and network diagrams were used to visualize the communication number and strength. In addition, the netVisual_bubble function was utilized to generate bubble charts, highlighting the significant ligand-receptor interactions between target cell clusters and other cell clusters, as well as emphasizing the key communication pathways between different cell types.

### 2.4 Ubiquitination scoring

A total of 1,349 ubiquitination-related genes were screened from the Genecards (https://www.genecards.org/) with the criterion of relevance score more than 5. The AUCell package was used to calculate the ubiquitination scores, and the ggviolin function was applied to plot violin plots.

### 2.5 High-dimensional weighted gene co-expression network analysis

We performed co-expression network analysis on the epithelial cells, using the “hdWGCNA” package (12). The “TestSoftPowers” function was used to determine the soft threshold, and its results were visualized using the “PlotSoftPowers” function. The co-expression network was constructed via the “ConstructNetwork” function. Additionally, the “PlotDendrogram” and “PlotKMEs” functions were employed to generate the dendrogram and gene ranking plot, respectively. Modules most relevant to ubiquitination were identified based on kME values.

### 2.6 Gene Ontology analysis

The “enrichGO” function was used to analyze the gene ontology (GO) functions of genes in the selected modules, with the screening criterion of q value less than 0.05. The “ggplot2” package was applied to visualize the enrichment results.

### 2.7 Differentially expressed genes

We used R package “Limma” to identify differentially expressed genes (DEGs) between involved sites of CD patients and intestinal tissues healthy control in the GSE75214 dataset. A P-value less than 0.05 and absolute log_2_ fold change more than 1 were considered statistically significant. In addition, the genes from the module most relevant to ubiquitination obtained by hdWGCNA were intersected with the DEGs to obtain candidate genes.

### 2.8 Screening of core ubiquitination-related genes

XGBoost, an integrated learning algorithm that uses decision trees as base learners, was applied to screen core genes. Nomogram, decision curve analysis, and ROC curve were used to evaluate the diagnostic value of core genes for CD.

### 2.9 Immune-related analysis and transcription factor prediction

The CIBERSORT algorithm was used to calculate immune cell scores. “Spearman” correlation analysis was performed to assess the correlations between core genes and immune cells, as well as immune checkpoint molecules, and the “pheatmap” function was used to draw the correlation heatmap. The ChEA3 database (https://maayanlab.cloud/chea3/) was utilized to predict the most closely related transcription factors that regulate the expression of core genes.

### 2.10 Cell culture

THP-1 cells were cultured in RPMI 1640 medium (Servicebio, G4531) Supplemented with 10% fetal bovine serum (Servicebio, G8003) and 0.05 mM β-mercaptoethanol (MedChemExpress, HY-Y0326), and incubated in a humidified atmosphere at 37°C with 5% CO_2_. THP-1 cells were differentiated into macrophages by treatment with 100 ng/ml phorbol 12-myristate 13-acetate (MedChemExpress, HY-18739) for 24 hours. Subsequently, cells were stimulated with 100 ng/ml lipopolysaccharide (MedChemExpress, L2880) and 20 ng/ml interferon-γ (Thermo Fisher Scientific, 300-02-20UG) for 48 hours to induce an inflammatory response. Three samples were used in each group.

### 2.11 Immunohistochemistry

Human colon tissue was fixed with 4% paraformaldehyde and embedded in paraffin. Paraffin sections were deparaffinized with xylene and rehydrated using a graded ethanol series. Antigen repair was performed by boiling the slices in EDTA antigen repair solution (Servicebio, G1039) for 30 minutes. Slices were placed in a 3% hydrogen peroxide solution (ANNJET) and incubated at room temperature in the dark for 25 minutes. Then, they were washed three times with PBS, each time for 5 minutes. Seal the sections with 3% bovine serum albumin at 37°C for 30 minutes, then incubate overnight with primary antibody at 4°C. The primary antibodies used are as follows: anti-IFITM3 (Proteintech, 1714-1-AP, 1:2000), anti-TAP1 (Proteintech, 68412-1-Ig, 1:500), and anti-PSMB9 (Proteintech, 67748-1-Ig, 1:300). After washing with PBS, incubate the slices with secondary antibodies (Servicebio, GB23303 or GB23301) at 37°C for 50 minutes. After washing with PBS, DAB was used for color development, and brown particle staining showed positive expression. The color development was terminated with tap water. Slices were counterstained with hematoxylin for 3 minutes (Servicebio, G1004), differentiated with hematoxylin differentiation solution (Servicebio, G1039) for a few seconds, rinsed with running water, and then stained with hematoxylin blue solution (Servicebio, G1040) and rinsed with running water. Dehydrate with graded ethanol, n-butanol, and xylene, and seal the film with sealing tape. Observe under a white light microscope and perform quantitative analysis using Image J software.

### 2.12 Real-time fluorescent PCR

RNA was extracted using the TransZol Up kit (TransGen Biotec, ET111-01-V2), and reverse transcription was performed with the Thermo Scientific RevertAid First Strand cDNA Synthesis Kit. Real-time Quantitative PCR was carried out in a Roche LightCycler 96 instrument using PerfectStart^®^ Green qPCR SuperMix (TransGen Biotec, AQ601-01-V2). The specific thermal cycling protocol consisted of an initial denaturation step at 94°C for 30 seconds, followed by 45 cycles of denaturation at 94°C for 5 seconds and annealing/extension at 60°C for 30 seconds. The specificity of all primer pairs was verified by melting curve analysis. β-actin was used as the internal reference gene. All primers used for RT-qPCR were synthesized by Tsingke Biotechnology (Hunan, China). The sequences of the primers used in this study are listed in the **Supplementary Material S3**.

### 2.13 Western blotting

Cells were lysed using SDS lysis buffer (Beyotime, P0013g). Protein concentration was measured with a BCA protein assay kit (Abbkine, KTD3001). Protein were separated by 12% sodium dodecyl sulfate-polyacrylamide gel electrophoresis and then transferred to a transfer membrane (Immobilon). After blocking with 5% non-fat milk for 1.5 hours, the membrane was incubated with the primary antibodies overnight at 4°C. The primary antibodies used were as follows: anti-IFITM3 (Proteintech, 1714-1-AP, 1:5000), anti-TAP1 (Proteintech, 68412-1-Ig, 1:5000), anti-PSMB9 (Proteintech, 67748-1-Ig, 1:5000), and anti-GAPDH (Proteintech, 60004-1-Ig, 1:100000). On the next day, the membrane was washed three times with TBST and incubated with secondary antibodies for 1 hour at room temperature. Detection and visualization of protein bands were performed using an ECL kit (Abbkine, BMU102) and Amersham ImageQuant 800 (Japan, Cytiva). Band intensities were quantified using Image J software.

### 2.14 Statistical analysis

The GSE134809 dataset was analyzed using the R packages “Seurat” and “SingleR”. The Mann-Whitney U test was used to analyze differences between the two groups. For evaluating core genes, receiver operating characteristic (ROC) curves and Nomogram plots were used to assess their diagnostic performance. Spearman correlation analysis was conducted to evaluate the correlations between core genes, immune cells, and immune checkpoint inhibitors. A p-value less than 0.05 was considered statistically significant.

## 3 Results

### 3.1 Cellular landscape of CD dissected by single-cell analysis

**Figure 1:**
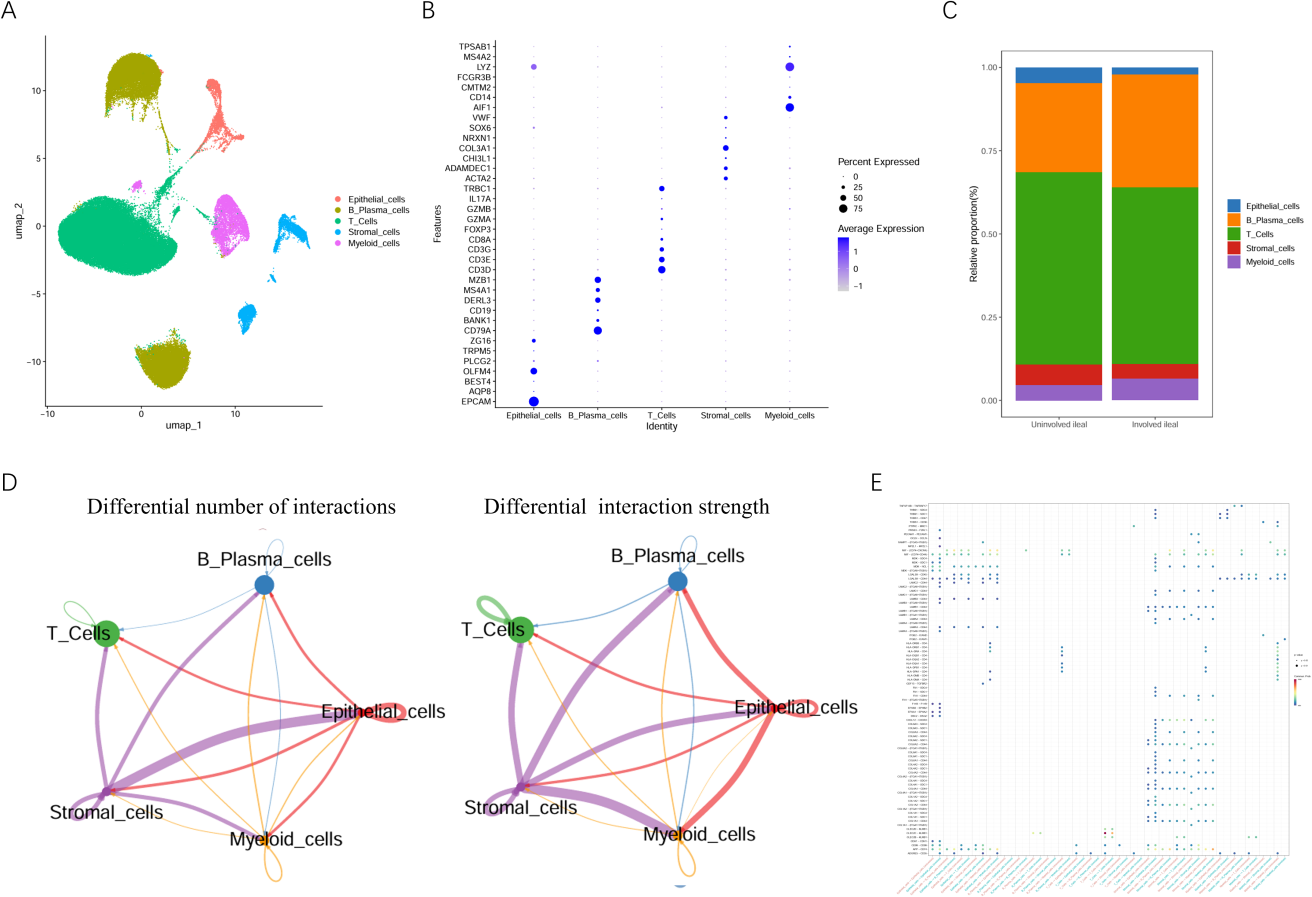
Identification of CD cell components and their relationships through single-cell sequencing database. **(A)** The UMAP diagram shows that the cells are divided into five groups; **(B)** The bubble plot displays the expression of marker genes of each cell type; **(C)** The proportion of various cell types in the uninvolved ileal and involved ileal tissues; **(D)** CellChat communication analysis elucidates the quantity and intensity of intercellular communication; **(E)** Bubble chart displays receptor-ligand pairs between cells.

To identify ubiquitination-related cell clusters, we performed single-cell transcriptomic analysis on different cell populations. All cells were divided into five distinct cell lineages based on the marker genes of various cell types and visualized using “UMAP” method (**Figure 1A**). The genes “EPCAM”, “AQP8”, “BEST4”, “OLFM4”, “PLCG2”, “TRPM5” and “ZG16” were used as the marker genes for epithelial cells. “CD79A”, “BANK1”, “CD19”, “DERL3”, “MS4A1” and “MZB1” as the marker genes for B plasma cells. “CD3D”, “CD3E”, “CD3G”, “CD8A”, “FOXP3”, “GZMA”, “GZMB”, “IL17A” and “TRBC1” as the marker genes for T cells; “ACTA2”, “ADAMDEC1”, “CHI3L1”, “COL3A1”, “NRXN1”, “SOX6” and “VWF” as the marker genes for stromal cells. “AIF1”, “CD14”, “CMTM2”, “FCGR3B”, “LYZ”, “MS4A2” and “TPSAB1” as the marker genes for myeloid cells (**Figure 1B**). We compared the proportions of various cell types in the uninvolved ileal and involved ileal segments, and found that the proportions of B plasma cells and myeloid cells were increased in the lesioned area, while the proportions of other cells were decreased (**Figure 1C**).

Additionally, CellChat analysis was conducted to characterize intercellular communications among these cell types. The results demonstrated that epithelial and stromal cells exhibited the highest interaction frequency at the quantitative level. In terms of communication intensity, stromal cells displayed robust interactions with all other cell populations (**Figure 1D**), suggesting their pivotal role in orchestrating the microenvironment of IBD-affected tissues. The ligand-receptor correlation map further revealed multiple ligand-receptor pairs mediating cross-talk between stromal and epithelial cells with other cell types (**Figure 1E**).

### 3.2 Screening of ubiquitination-related genes

**Figure 2:**
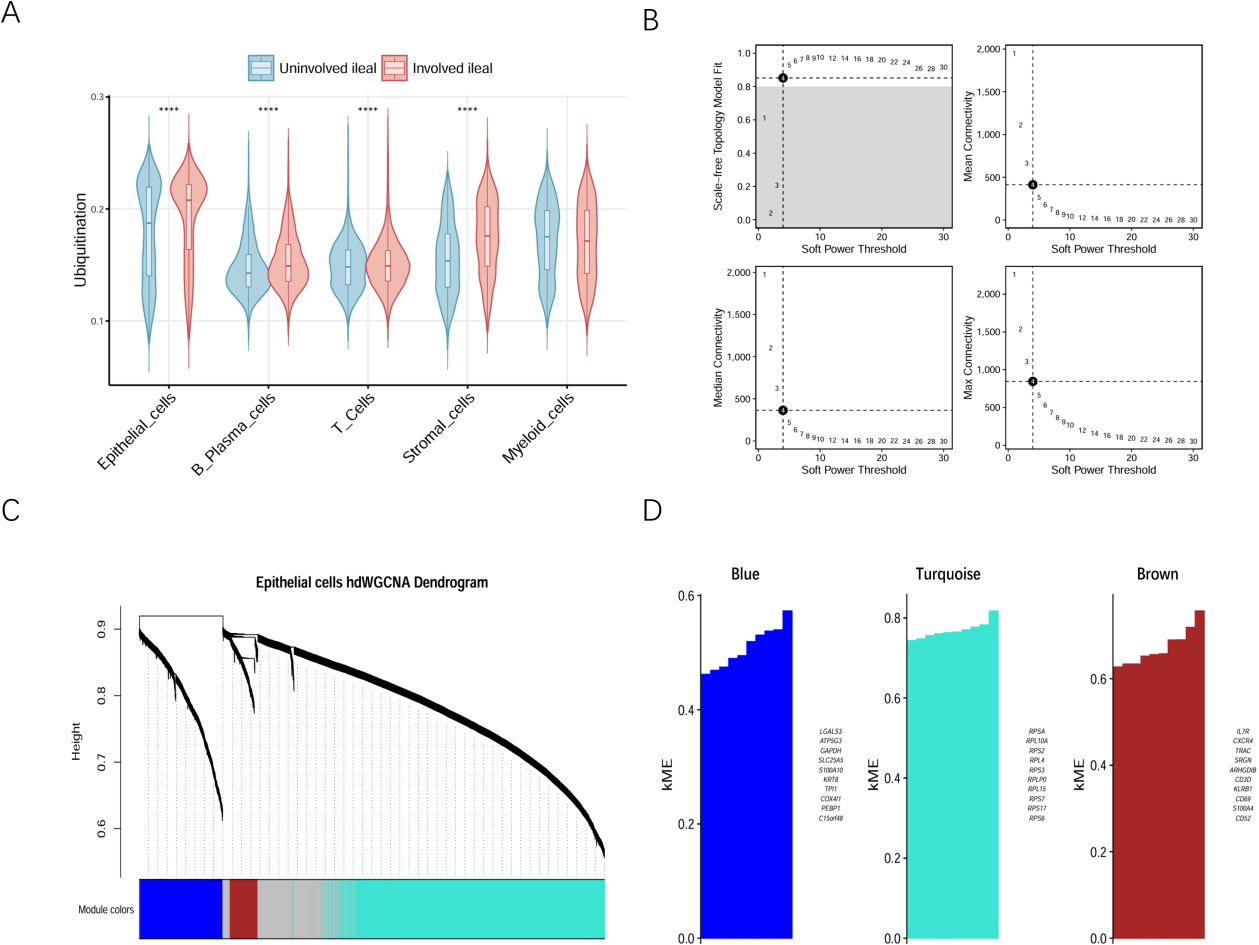
Screening of genes related to ubiquitination. **(A)** Ubiquitination scores for various cell types; **(B)** High dimensional weighted edge expression network analysis of single-cell data determined a soft threshold of four; **(C)** Dendrogram of gene modules; **(D)** The kME value of three modules.

**Figure 3:**
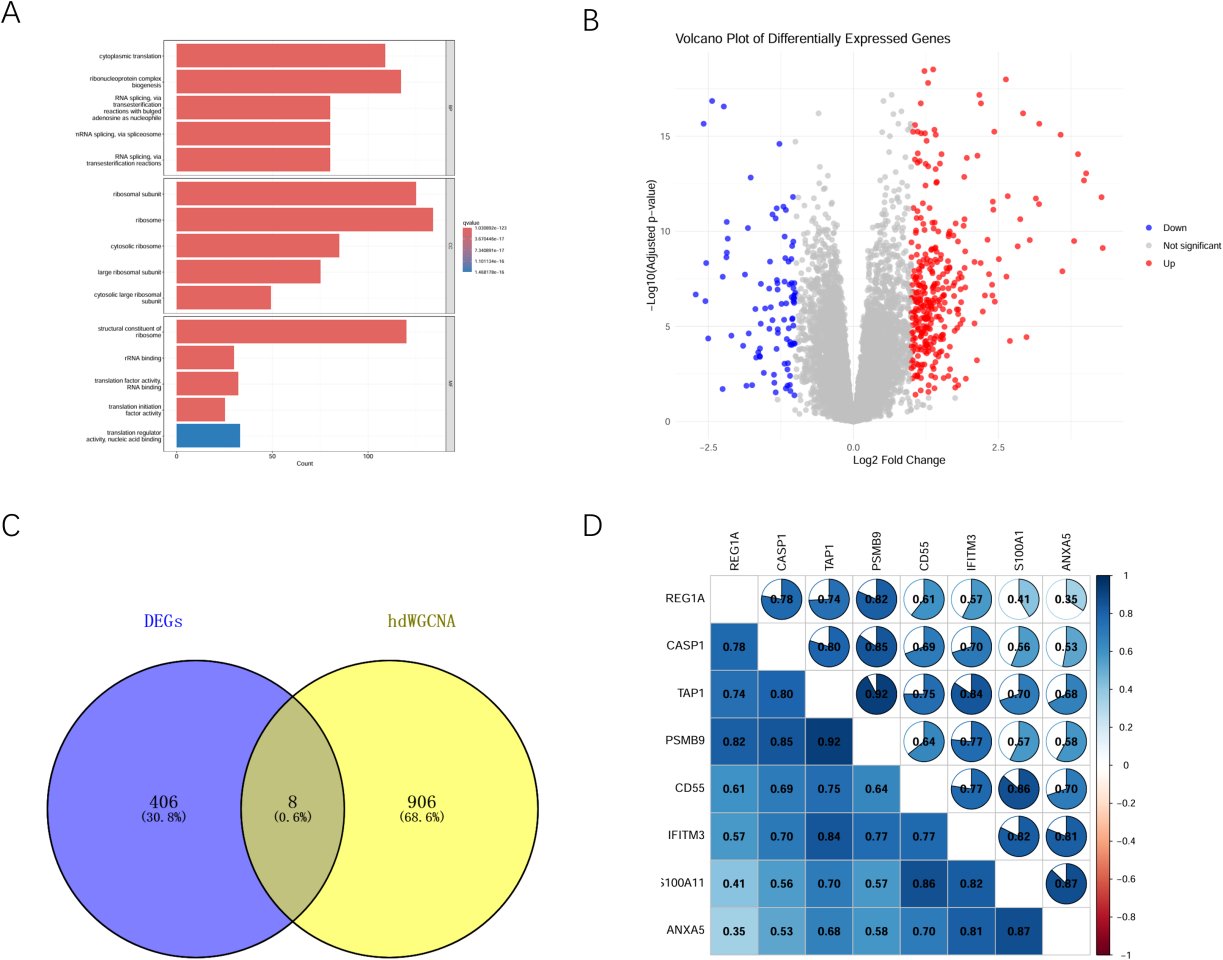
Screening of differentially expressed ubiquitination-related genes. **(A)** Enrichment analysis of Turquoise module genes with kME greater than 0.3; **(B)** Volcanic diagram of differentially expressed genes; **(C)** Venn diagram identifying ubiquitination-related DEGs; **(D)** Correlation analysis of selected ubiquitination-related gene.

The ubiquitination scores of each cell type in the involved and uninvolved regions were compared. The results showed that the ubiquitination score was higher in the involved region than in the uninvolved region, and the ubiquitination score of epithelial cells was higher than that of other cells (**Figure 2A**). Therefore, epithelial cells were selected for further analysis. The hdWGCNA was used to identify genes related to ubiquitination. Based on a soft threshold of four, the genes were divided into three similar co-expression functional modules: Blue, Turquoise, and Brown modules (**Figures 2B–D**). As shown in **Figure 2D**, epithelial cells had the highest correlation with genes in the Turquoise module, indicating that these genes might be involved in the ubiquitination process. The Turquoise module contained 914 genes with a correlation coefficient greater than 0.3. GO enrichment analysis revealed that these genes were mainly related to biological processes such as cytoplasmic translation and ribonucleoprotein complex biogenesis, as well as ribosomal component composition (**Figure 3A**). Based on the GSE75214 dataset, 414 DEGs were identified. By taking the intersection of genes in the Turquoise module screened by hdWGCNA and the DEGs, eight genes were obtained (**Figures 3B, C**). Correlation analysis of these eight genes showed that their expressions were all positively correlated with each other (**Figure 3D**).

### 3.3 Diagnostic value of core genes

**Figure 4:**
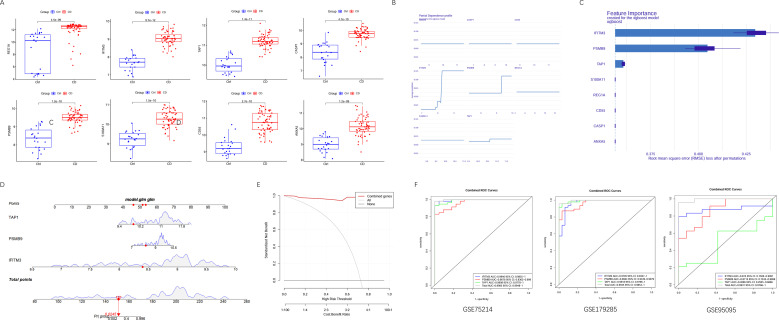
Machine learning screening for ubiquitination-related core genes. **(A)** Box plot of ubiquitination-related genes expression in CD and healthy patients; **(B, C)** XGBoost used for screening ubiquitination-related core genes. **(D–F)** Nomogram, decision curve analysis, and ROC curve were used to evaluate the diagnostic value of ubiquitination-related core genes for CD.

Expression analysis showed that all eight genes were significantly upregulated in CD patients compared with the control group (**Figure 4A**). The XGBoost algorithm and the increment of the root mean square error was used to identify three core genes that had a greater impact on the model results, namely IFITM3, PSMB9, and TAP1 (**Figures 4B, C**). A diagnostic nomogram was constructed using core genes (**Figure 4D**). The decision curve analysis showed that the net benefit of the diagnostic model was greater than 0.8, indicating high sensitivity and specificity of the diagnostic model (**Figure 4E**). ROC curve analysis demonstrated that the diagnostic model constructed based on the core genes exhibited strong discriminative ability across GSE179285 and GSE95095 dataset (**Figure 4F**).

### 3.4 Immune correlation analysis

**Figure 5:**
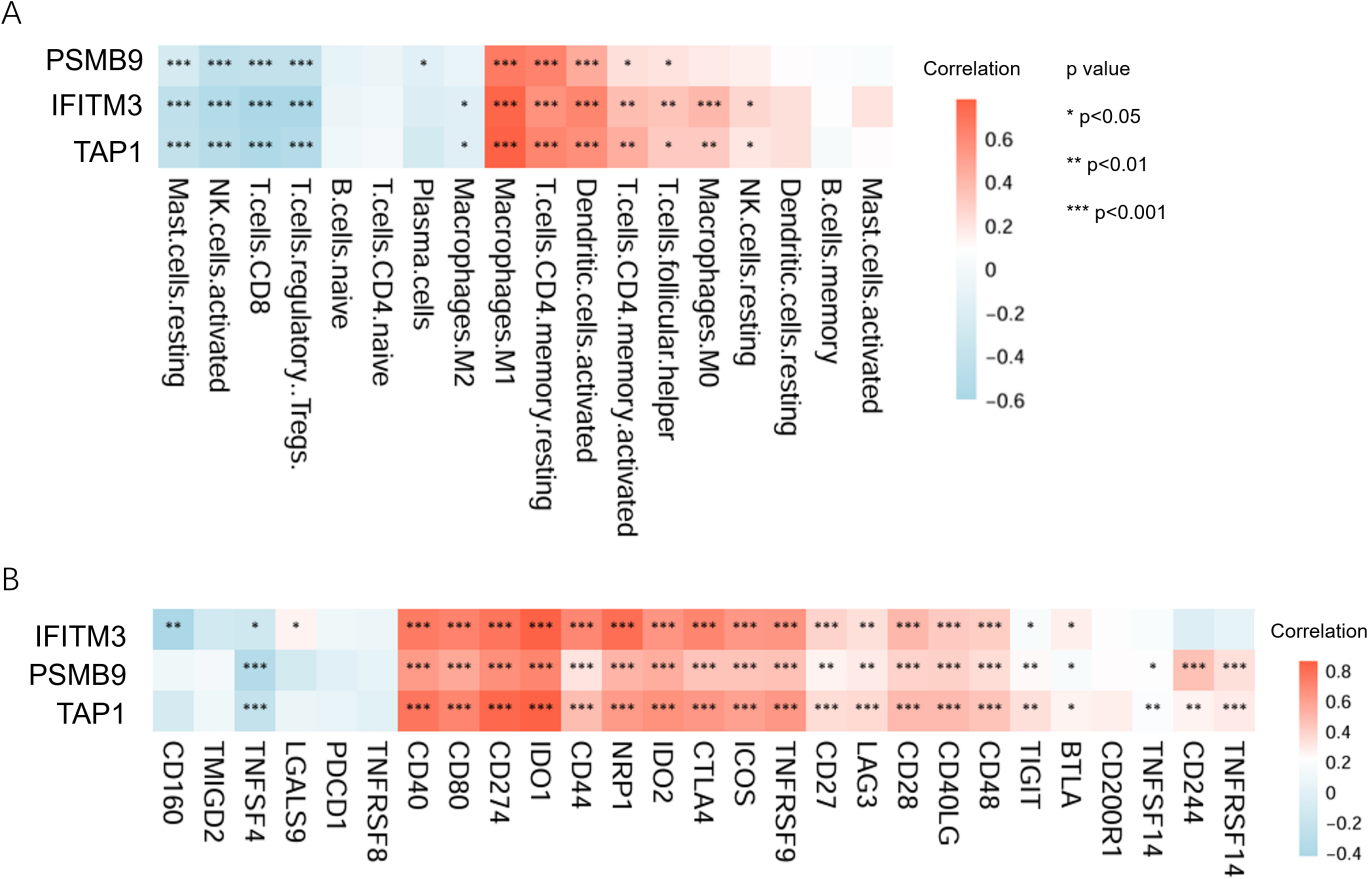
Immune related analysis of ubiquitination-related core genes. **(A)** The heatmap shows the correlation between ubiquitination-related core genes and immune cells. **(B)** The heatmap shows the correlation between ubiquitination-related core genes and immune checkpoint molecules.

We found that the three core genes were significantly correlated with activated immune cells in the inflammatory microenvironment, such as M1 macrophages, resting memory CD4+ T cells, and activated dendritic cells (**Figure 5A**). Additionally, they exhibited positive correlations with immune checkpoints including CD40, CD80, and CD274 (**Figure 5B**).

### 3.5 Transcription factor prediction and expression of core genes

**Figure 6:**
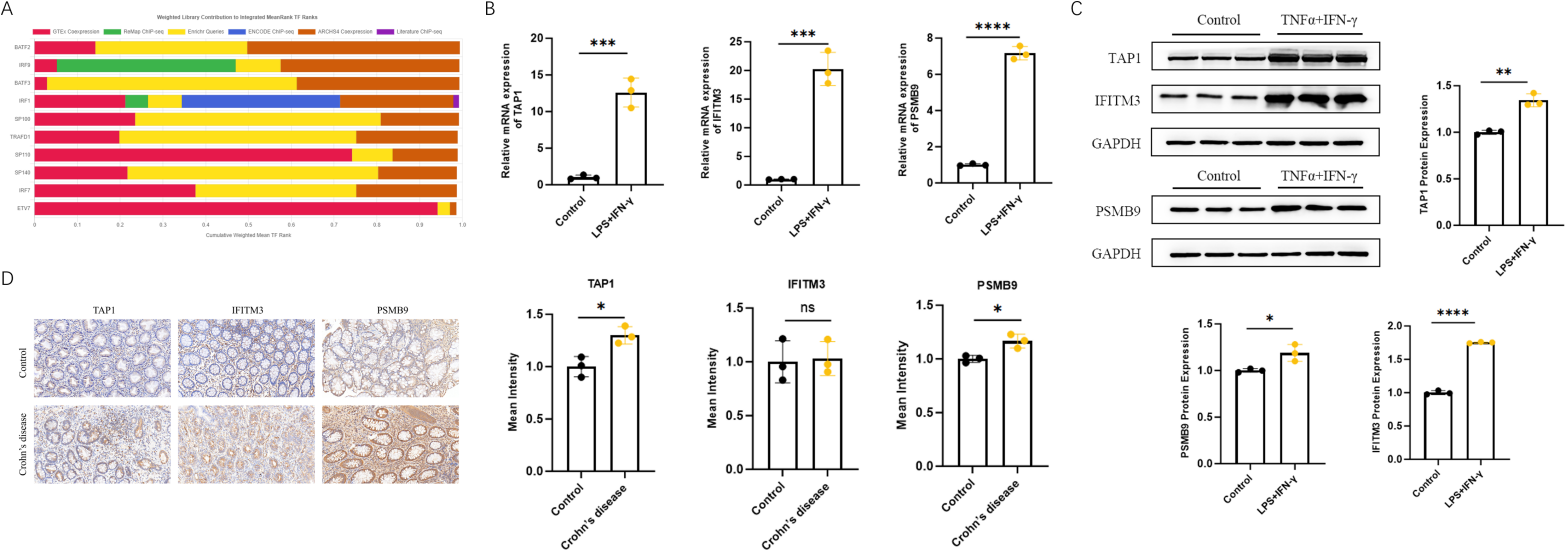
Expression of ubiquitination-related core genes IFITM3, PSMB9, and TAP1 in CD. **(A)** Prediction of transcription factors regulating ubiquitination-related core genes using hEA3 database; **(B, C)** qPCR and WB were used to detect the expression levels of ubiquitination-related core genes in THP-1 cells induced by LPS and IFN-γ. ^ns^p>0.05, *p<0.05, **p<0.01; ***p<0.001, ****p<0.0001; **(D)** Immunohistochemistry displays the expression of ubiquitination-related core genes in human tissue specimens.

We predicted transcription factors that regulate the core genes, including BATF2 and IRF9 (**Figure 6A**). After treating THP-1 cells with LPS and IFN-γ, we observed a significant increase in the expression of the three core genes under inflammatory conditions (**Figures 6B, C**). The original images were presented in **Supplementary Material S4**. Immunohistochemical staining was performed on intestinal tissue samples from lesioned sites of CD patients and control individuals, revealing that the expression levels of TAP1 and PSMB9 were significantly elevated in CD patients, whereas there was no significant difference in the expression level of IFITM3 between the two groups (**Figure 6D**).

## 4 Discussion

CD is a subtype of IBD, characterized by recurrent intestinal inflammation. Globally, the prevalence of CD has been steadily increasing (13). CD is associated with factors such as environment, intestinal microbiota, genetics, and immunity. The advent of biological agents has significantly improved the therapeutic efficacy of CD and enhanced patients’ quality of life, but it cannot achieve a complete cure. Research is constantly exploring the pathogenesis of CD to identify new therapeutic targets and treatment methods.

Ubiquitination and deubiquitination are post-translational modifications of proteins. The process of ubiquitin modification mainly involves three key modifying enzymes. A number of studies have identified multiple genes related to ubiquitin-modifying enzymes, including USP1, USP3, OTUD3, and so on, as susceptibility gene loci for IBD, which implies that ubiquitin-modifying enzymes are involved in the pathophysiology of IBD (14). Ubiquitin-modifying enzymes influence the onset and development of IBD by regulating intestinal barrier function (15), innate immune responses (9), and adaptive immune responses (16). TRIM8-mediated CAPN2 ubiquitination drives ZBP1-dependent PANoptosis in intestinal epithelial cells, thereby exacerbating CD (17). N4BP3 promotes the release of inflammatory factors in IBD by mediating K63-linked RIPK2 ubiquitination (18). Deubiquitinating enzymes are also closely related to inflammation in IBD. Studies have shown that the deficiency of deubiquitinating enzyme Josephin domain containing 2 exacerbates colonic inflammation (19). Another deubiquitinating enzyme, ovarian tumor deubiquitinase 1, has an inhibitory effect on RIPK1-mediated NF-κB activation, thereby reducing intestinal inflammation (20). Therefore, ubiquitination is involved in the inflammatory response process in IBD through multiple pathways.

In this study, single-cell transcriptomics analysis was employed to deeply dissect the cellular landscape of IBD. The study found that the proportions of B plasma cells and myeloid cells in the lesioned ileum were significantly increased, revealing specific changes in cellular composition within the IBD microenvironment. CellChat analysis further identified the pivotal role of epithelial cells and stromal cells in intercellular communication. Using the hdWGCNA algorithm, it was determined that genes in the Turquoise module of epithelial cells are closely associated with the ubiquitination process, and ultimately, the core genes IFITM3, PSMB9, and TAP1 were screened out. These genes not only show significantly elevated expression in CD patients, but the diagnostic model constructed based on them also exhibits good sensitivity and specificity. The diagnosis of CD requires a comprehensive judgment based on clinical symptoms, medical history, and some examinations. Similarly, IFITM3, PSMB9, and TAP1 can assist in diagnosis of CD. Additionally, the core genes are closely related to activated immune cells and immune checkpoints under inflammatory conditions, providing new clues for the pathogenesis and diagnosis of IBD.

Our study found that the expression levels of PSMB9, and TAP1 are elevated in CD patients, which is consistent with the findings of Feng Wu et al. Their study showed that, compared with the healthy control group, the differentially expressed genes in CD samples are related to IFN-γ-induced TH1 processes (IFITM1, IFITM3, STAT1, and STAT3) and antigen presentation (TAP1, PSME2, and PSMB8) (21). We found no significant increase in IFITM3 in CD patient specimens compared to healthy controls using immunohistochemical staining. However, analysis using the GEO database containing 59 CDs and 22 healthy controls revealed a significant increase in IFITM3 in the intestinal tissue of CD patients. Western blot analysis on cells also showed a significant increase in IFITM3 protein under inflammatory conditions. In addition, Feng Wu et al. aimed to demonstrate the gene expression pattern of IBD. Their study included 4 healthy individuals and 7 CD patients, and found that IFITM3 was significantly elevated in CD patients (21). Therefore, based on the GEO database, Western blotting, and published literature, we believe that the lack of significant differences in IFITM3 on human tissue specimens is due to individual differences and small sample sizes. The interferon induced transmembrane protein 3 (IFITM3) is a transmembrane protein with multiple functions, including roles in viral infection, Alzheimer’s disease, and cancer (22, 23). The expression level of IFITM3 is upregulated in various activated immune cells, including macrophages, dendritic cells, T cells, and B cells. A variety of pro-inflammatory cytokines such as interferon, IL-1, and IL-6 can increase the expression of IFITM3 (24). Our study suggests that IFITM3 is related to ubiquitination in IBD, but the role of IFITM3 in the occurrence and development of IBD has not been reported yet, and further in-depth research is still needed.

Proteasome 20S subunit beta 9 (PSMB9) encodes a catalytic subunit β1i of the immunoproteasome, which assembles with β2i (PSMB10) and β5i (PSMB8) to form the core particle of the immunoproteasome (25). The immunoproteasome is a subtype of proteasome. It can not only effectively clear proteins in both ubiquitin-dependent and ubiquitin-independent manners through its enhanced proteolytic activity (26), but also regulate the production of pro-inflammatory cytokines as well as the differentiation and proliferation of T cells (27). In recent years, the immunoproteasome has emerged as a potential drug target for various autoimmune diseases, such as rheumatoid arthritis (28) and IBD (29, 30). PSMB9 is involved in cleaving antigenic peptides and presenting them on the cell surface, thereby activating cellular immunity. It is also involved in protein degradation, maintaining protein homeostasis and Th cell differentiation (31). PSMB9 plays a crucial role in various diseases, including neurodegenerative diseases, malignant tumors, and autoimmune diseases (31, 32). Viviana Scalavino et al. also found that elevated levels of PSMB9 were observed in colonic samples from patients with acute IBD compared with those in the IBD remission group and the control group (33). Inhibition of PSMB9 under inflammatory conditions can induce E3 ligase TRIM31-mediated NLRP3 degradation, reduce the formation of NLRP3 inflammasomes in TNFα-stimulated intestinal epithelial cells, and thereby alleviate NLRP3 inflammatory responses that trigger intestinal barrier disruption (34, 35).

Transporter associated with antigen processing 1 (TAP1) is a membrane-bound protein. TAP1 and TAP2 form a heterodimeric complex, which is mainly located in the endoplasmic reticulum, with some present on the Golgi membrane. The TAP complex can assist in the transmembrane transport of intracellular polypeptides into the endoplasmic reticulum. In the endoplasmic reticulum, it forms a peptide loading complex (PLC) together with other components. The PLC loads high-affinity polypeptides onto adjacent major histocompatibility complex class I (MHC-I) molecules, and then the MHC-I molecules present the antigenic peptides on the cell surface. These displayed polypeptides are subsequently recognized by CD8+ cytotoxic T lymphocytes (CTLs) to initiate an immune response (36). TAP1 is closely related to the processing and presentation of endogenous antigens. A number of studies have pointed out that TAP1 gene polymorphisms are associated with autoimmune diseases such as ankylosing spondylitis and psoriasis (37, 38). Our study shows that the expression of TAP1 is increased in CD patients, which is consistent with the research results of Lili Yang et al. (39). Ziyu Liu et al. found that the TAP1 gene is a strong predictor with the highest diagnostic value for IBD among endoplasmic reticulum stress-related genes (area under the curve equals 0.941) (40).

Our study has several limitations. First, the diagnostic model established in this study is derived from database data, and its generalizability needs to be validated in a larger and more diverse cohort. Second, while transcriptomic and single-cell RNA sequencing data were utilized, the study lacks integration with other omics approaches, such as proteomics and metabolomics. Future research should incorporate multi-omics data to comprehensively elucidate the pathogenesis of CD and enhance the predictive performance of the model. Besides, although IFITM3, PSMB9, and TAP1 have been identified as potential biomarkers for CD, their roles in CD pathogenesis have rarely been reported, necessitating further experimental validation. Additionally, we have not established the specificity of these three genes as CD-specific markers in the present study. Future work will involve large-scale validation with samples of ulcerative colitis, infectious enteritis, and other intestinal inflammatory conditions to further clarify their specificity for CD. Finally, the potential intercellular communication patterns predicted in this work hold theoretical reference value but require further experimental validation. In future research, we will perform experiments including *in vitro* co-culture and receptor-ligand binding assays to further confirm the authenticity of these interactions.

## 5 Conclusions

We set up a novel diagnostic model for CD patients based on ubiquitination-related genes IFITM3, PSMB9, and TAP1, pinpointing it as a promising diagnostic model.

## Data Availability

The original contributions presented in the study are included in the article/**Supplementary Material**. Further inquiries can be directed to the corresponding authors.

## Supplementary material

Supplementary Material S1 Baseline data of patients in two groups.

Supplementary Material S2 Basic information about involved GEO datasets.

Supplementary Material S3 Primer sequences for ubiquitination-related core genes.

Supplementary Material S4 Original blots of Western blot analysis of IFITM3, PSMB9, and TAP1 in THP-1 cells.
