# Supplementary figures and images for: Novel ubiquitination-related biomarkers for Crohn’s disease identified by multi-omics study and experimental validation
Source: Front Immunol. 2025 Dec 5;16:1687606. doi: 10.3389/fimmu.2025.1687606 (PMC12714605; doi:10.3389/fimmu.2025.1687606)

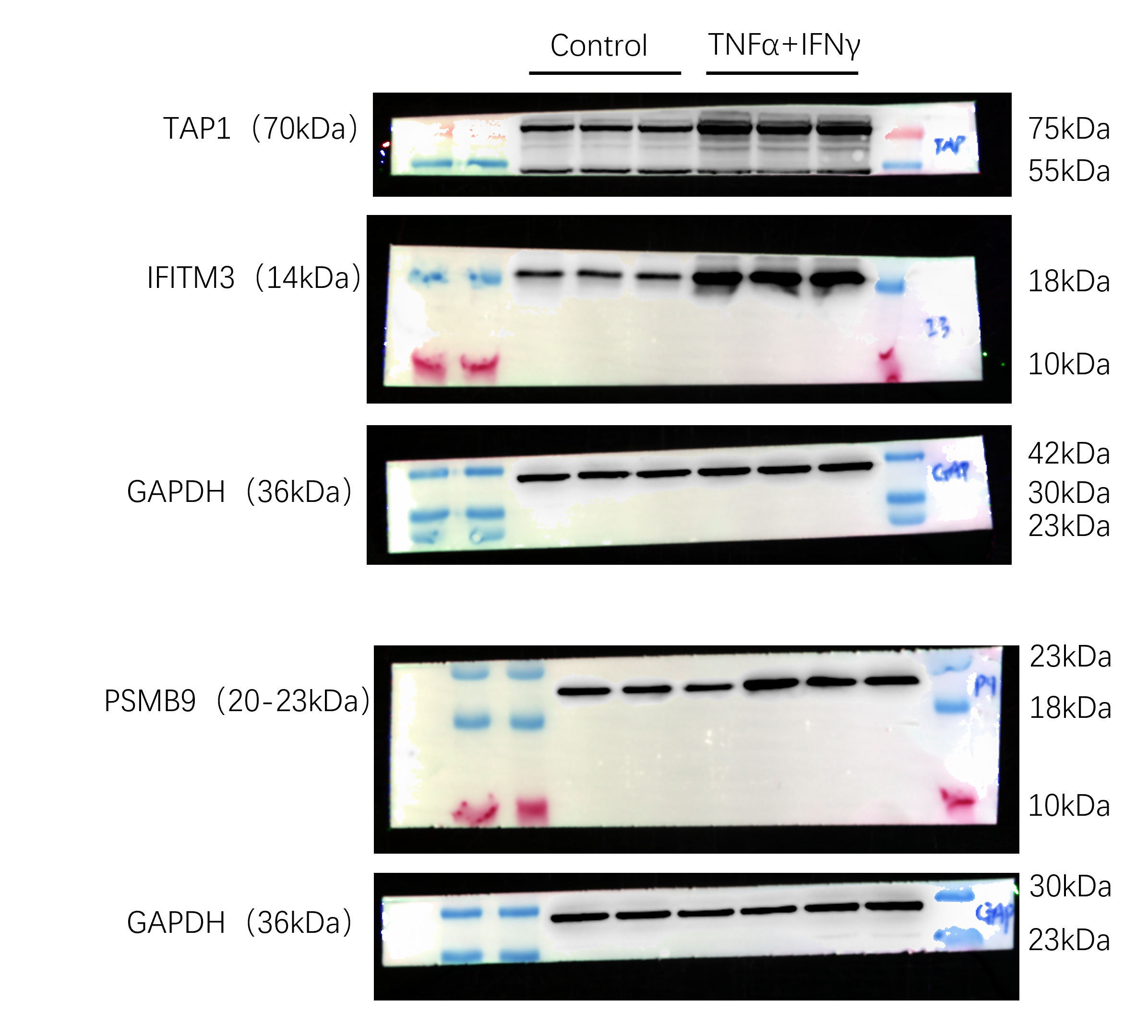

Supplement: Supplementary Material S1 — Baseline data of patients in two groups. [file Image1.tif]
