# Supplementary material for: Novel ubiquitination-related biomarkers for Crohn’s disease identified by multi-omics study and experimental validation
Source: Front Immunol. 2025 Dec 5;16:1687606. doi: 10.3389/fimmu.2025.1687606 (PMC12714605; doi:10.3389/fimmu.2025.1687606)
Supplement: Supplementary Material S2 — Basic information about involved GEO datasets. [file Table1.docx]

Supplemental material 1. Baseline data of patients in two groups

|  | Characteristics | CD | Intestinal polyp | *P*-value |
| --- | --- | --- | --- | --- |
| IFITM3 | Age (yr) | 42.67±1.528 | 41.33±18.15 | 0.9052 |
|  | Sex (n) |  |  | >0.999 |
|  | Male | 2 | 2 |  |
|  | Female | 1 | 1 |  |
| PSMB9 | Age (yr) | 44±13.08 | 40.33±17.21 | 0.7835 |
|  | Sex (n) |  |  | >0.999 |
|  | Male | 0 | 1 |  |
|  | Female | 3 | 2 |  |
| TAP1 | Age (yr) | 47±10.82 | 46.67±21.13 | 0.9818 |
|  | Sex (n) |  |  | >0.999 |
|  | Male | 1 | 1 |  |
|  | Female | 2 | 2 |  |
