# Supplementary material for: Novel ubiquitination-related biomarkers for Crohn’s disease identified by multi-omics study and experimental validation
Source: Front Immunol. 2025 Dec 5;16:1687606. doi: 10.3389/fimmu.2025.1687606 (PMC12714605; doi:10.3389/fimmu.2025.1687606)
Supplement: Supplementary Material S3 — Primer sequences for ubiquitination-related core genes. [file Table2.docx]

Supplemental material 2. Basic information about involved GEO datasets.

| Datasets | Crohn’s disease | | |  | Control | | | Sequencing platforms |
| --- | --- | --- | --- | --- | --- | --- | --- | --- |
|  | Total | Ileal | Colon |  | Total | Ileal | Colon |  |
| GSE134809 | 11 | 11 | 0 |  | 11 | 11 | 0 | [GPL18573](https://www.ncbi.nlm.nih.gov/geo/query/acc.cgi?acc=GPL18573) |
| GSE75214 | 59 | 51 | 8 |  | 22 | 11 | 11 | [GPL6244](https://www.ncbi.nlm.nih.gov/geo/query/acc.cgi?acc=GPL6244) |
| GSE20881 | 73 | 17 | 56 |  | 59 | 6 | 53 | [GPL1708](https://www.ncbi.nlm.nih.gov/geo/query/acc.cgi?acc=GPL1708) |
| GSE95095 | 24 | NA | NA |  | 12 | NA | NA | [GPL14951](https://www.ncbi.nlm.nih.gov/geo/query/acc.cgi?acc=GPL14951) |
