# Supplementary material for: Novel ubiquitination-related biomarkers for Crohn’s disease identified by multi-omics study and experimental validation
Source: Front Immunol. 2025 Dec 5;16:1687606. doi: 10.3389/fimmu.2025.1687606 (PMC12714605; doi:10.3389/fimmu.2025.1687606)
Supplement: Supplementary Material S4 — Original blots of Western blot analysis of IFITM3, PSMB9, and TAP1 in THP-1 cells. [file Table3.docx]

Supplemental material 3. Primer sequences

| Gene | Species | Sequence (5’ → 3’) |
| --- | --- | --- |
| ACTB | Human | F: GAGCACAGAGCCTCGCCTTT |
|  |  | R: TCATCATCCATGGTGAGCTGG |
| IFITM3 | Human | F: TGCTGATCTTCCAGGCCTATG |
|  |  | R: GGCGAGGAATGGAAGTTGGA |
| PSMB9 | Human | F: GCACCAACCGGGGACTTAC |
|  |  | R: CACTCGGGAATCAGAACCCAT |
| TAP1 | Human | F: TGCCCCGCATATTCTCCCT |
|  |  | R: CACCTGCGTTTTCGCTCTTG |
